# Toward equitable payment for inclusive participation

**DOI:** 10.1017/cts.2023.5

**Published:** 2023-02-27

**Authors:** Justin X. Moore, LaKesha N. Anderson, Cynthia Li, Reginald D. Benson, Alejandra Garcia Rychtarikova, Lillie D. Williamson, Leslie E. Wolf, Ebony B. Whisenant, Erin Roark, Samantha R. Jones, Christy J.W. Ledford

**Affiliations:** 1 Cancer Prevention, Control, & Population Health Program, Georgia Cancer Center, Department of Medicine, Medical College of Georgia at Augusta University, Augusta, GA, USA; 2 Department of Medicine, Uniformed Services University, Bethesda, MD, USA; 3 Medical College of Georgia, Augusta University, Augusta, GA, USA; 4 Department of Family and Community Medicine, Medical College of Georgia at Augusta University, Augusta, GA, USA; 5 Department of Communication Arts, University of Wisconsin-Madison, Madison, WI, USA; 6 Center for Law, Health & Society, Georgia State University College of Law, Atlanta, GA, USA; 7 Katherine Reese Pamplin College of Arts, Humanities, and Social Sciences at Augusta University, Augusta, GA, USA

*Oppressed peoples are always being asked to stretch a little more, to bridge the gap between blindness and humanity.*

– Audre Lorde

Because of discrimination and exclusion, historically marginalized populations continue to experience disparities in health, wealth, education, housing, and employment and have been described as “vulnerable” in the research context [1]. Researchers and institutional review boards (IRBs) persistently assert that payments may be “unduly influential” and undermine the voluntariness of consent [2–4]. However, this potentially unwarranted ethical concern may result in imbalanced payments. Participant payment structures that calculate reimbursement based on participant work income assign relative value to participation, providing participants with lower income lower financial payment [5]. Underrepresentation in research may be exacerbated by payment structures that deter participation, such as requiring participants to share social security numbers or other identifying information [6].

Although translational science demonstrates that payments themselves are successful in increasing recruitment [7], there is little consensus on how to ethically and inclusively structure payments. Payment structure is more than simply the amount of payment. Bioethicists conceptualize participant payments in four ways: appreciation, reimbursement, compensation, and incentive [2,8]. A 2005 analysis of 467 IRB-approved protocols offering payments to research participants found protocols rarely provide the rationale for payment [3].

To better understand current payment practices, our team conducted a content analysis of four journals (*Annals of Family Medicine, Annals of Internal Medicine, American Journal of Emergency Medicine,* and *American Journal of Obstetrics and Gynecology*), which publish clinical and translational research to inform clinical practice. Among 519 articles reviewed, 203 studies included human participants whose participation ranged from surveys or interviews to multiple blood draws and medical procedures. Only 5.4% (11 of 203) described a payment for research participation; and of these, only 9 provided information regarding payment types and structure (Table 1). Among papers that disclosed payment information, payment detail varied, see Table 2. Payments were more frequently given to healthcare workers than community or clinical participants, which may indicate a transactional approach. This finding directly diverges from the recommendation that payments “should reflect the general value of the time and burdens associated with the study” rather than factors like participant earning potential [2].

**Table 1.** Characteristics of published studies that enrolled human participants

		Total sample (*n* = 203)	Studies that disclosed participant payments (*n* = 11)
Journal*	*Annals of Family Medicine*	57 (28.1%)	7 (63.6%)
	*Annals of Internal Medicine*	19 (9.4%)	0
	*American Journal of Emergency Medicine*	55 (27.1%)	2 (18.2%)
	*American Journal of Obstetrics and Gynecology*	72 (35.5%)	2 (18.2%)
Funding source	Federal or state	93 (45.8%)	9 (81.8%)
	Private foundation or not-for-profit organization	30 (14.8%)	1 (9.1%)
	Commercial organization, such as drug or device manufacturer	5 (2.5 %)	0
	Other, including multiple categories	17 (8.4%)	1 (9.1%)
	No funding acknowledged	58 (28.6%)	0
Recruitment setting and participants	Clinical/patients	165 (81.3%)	2 (18.2%)
	Community members	12 (5.9%)	2 (18.2%)
	Hospital or clinic employees	20 (9.9%)	4 (36.4%)
	Online	6 (3%)	3 (27.3%)
Specified vulnerable populations**	No	194 (95.6%)	9 (81.8%)
	Yes	9 (4.4%)	2 (18.2%)

* The sampling frame included the first six issues of 2017–2019 for three journals (*AFM, AIM, AJOG*) and the first three issues 2017–2019 for *AJEM*.

** Vulnerable populations included: “women (in the context of diagnosing coronary heart disease),” “women who underwent fetoscopic laser therapy for twin-twin transfusion syndrome,” “suicide attempters,” “socioeconomically deprived areas,” “patients with depression,” “older adults,” “Community Health Centers,” “Black pregnant women,” and “pregnant smokers.” Notably, some papers described populations that our research team would have qualified as vulnerable, but the published papers did not use these terms or similar concepts, such as male youths who had been found guilty of serious criminal offense or women seeking abortion.

**Table 2.** Full description of participant payments

“…participants …received an honorarium for their participation.”
“At the end of the study, participants received a … $10 gift card.”
**“**Each participant received a gift card in return for participation in the study.”
“Panel members … receive incentives using a point system.”
“Panelists…receive compensation in points that can be redeemed for cash or goods…Compensation for participation was initially the cash equivalent of $4 for phone mode and $2 for online surveys… To encourage survey response in the second and third waves of data collection, compensation increased to the cash equivalent of $10 for both survey modes.”
“Participants did not receive financial compensation but they were offered points by the market research company, which can be accrued over time and redeemed for vouchers or for entering prize drawings.”
“Participants received $10 for completing the study survey and were entered into a lottery drawing for $100. In addition, the research team donated $200 to a local outreach effort of the clinic’s choice to clinics with 90% participation.”
“Participants were informed they would be paid $40 for up to 2 hours of their time.”
“Patients received approximately $15 cash equivalent in Nielsen points.”
“Residents… were given a gift card for $10 if they completed the study.”
“Respondents received a check for $100 by mail.”

Two conclusions can be drawn from these findings: 1) studies are not publishing payment information or 2) studies are underutilizing payments in recruitment. Clinical and translational science research studies should adopt more rigorous reporting guidelines regarding payment of research participants so we can determine which of these conclusions is correct.

Failure to publish payment information prevents us from understanding the use of payment in research and whether it is effective and equitable. Standardized clinical research guidelines increase reproducibility and enable fellow researchers to better understand methodological approaches. A standardized approach to describing payments may provide utility in contextualizing a study’s findings. Current guidelines such as the Consolidated Standards of Reporting Trials (CONSORT) do not include reporting of participant payments. We specifically recommend that researchers describe four features of payment structure: amount, rationale, mechanism, and timing. Our recommendation extends the 2019 Secretary’s Advisory Committee on Human Research Protections recommendation [9] from justification in IRB protocols to presenting the rationale in dissemination activities. Standardized dissemination could facilitate much needed research on investigator practices of paying research participants. Such information could lead to more ethical, equitable treatment of research participants and trust in the research enterprise.

To better understand the inclusion of populations, studies should also describe limitations of the study’s payment structure. None of the studies here described how the process of payment, such as collecting social security numbers, excludes vulnerable populations. This practice limits inclusion of populations who do not have access to federal identification systems, such as homeless individuals or undocumented workers. Vulnerable populations already have higher levels of mistrust in medical research [10]. Requiring collection of personal information limits inclusion of populations whose distrust of systems, informed by historic research abuses such as the US Public Health Service Tuskegee Syphilis Study [11], regulates how they disclose personal information.

Underutilization of payments in recruitment may slow research progress, but if, as our sample suggests, patient and community participants are least likely to receive payment, these practices may undermine efforts for more inclusive research. Mistrust is not only an antecedent of research participation but also a potential outcome. How participants feel about payment structures, including underpayment, may reinforce some of the ideas and beliefs that underlie mistrust (e.g., mistreatment, being used, etc.).

Given the historical context and untrustworthy behavior by US public health systems to vulnerable groups, we must be cognizant of how researchers are perceived by populations of interest for recruitment [10]. Critical approaches to health scholarship have called for researchers to interrogate the ways in which we engage with persistently marginalized communities, encouraging consideration of the needs and criteria that are important to communities [12]. Researchers should not unilaterally assign value to participation, particularly since perceptions of the amount of fair payment varies by race and ethnicity [13]. Community consultation and partnership not only will help determine fair payment that facilitates participation by providing reimbursement, adequate compensation, and incentives [2] but also what creates equitable, reciprocal relationships with communities [14]. The inclusion of community members is particularly important for communities who have experienced mistreatment and may continue to have fraught relationships with medical research [10].

The appropriateness of compensation and judgments of inconvenience or disruption can differ depending on the cultural values and perspectives of participants [15]. For instance, the same monetary value given in cash versus gift cards has implications for use. Festinger and Dugosh found that individuals given cash were more likely to pay for transportation and bills, whereas participants given gift cards were more likely to purchase household items [16]. Community input and inclusion in creating the payment structure may ensure that participants receive payments in a meaningful and useful way. It may also reduce the imperious stereotype of researchers.

Due to the dearth of information about payment processes, it is unclear the extent to which researchers are engaging in these activities or how they have done so effectively. Future research should further elaborate on participant payments and consider participants as co-researchers [17] in human subjects research as a mechanism toward greater equity in research.
